# The Treatment of Cough

**Published:** 1906-04

**Authors:** Chas. L. Ashley


### ABSTRACTS.

---

### The Treatment of Cough

By CHAS. L. ASHLEY. M. D., Wilkesbarre, Pa.

[*Medical Review of Reviews.*]

How many of us stop to think of the systematic disturbances produced by an ordinary prescription for a bronchitis or simple irritation with cough? How many of us think of the depressing effects of the opiates in continued use? What is the cause of the headache, the sick stomach, the diarrhea, the constipation and numerous other complications? Well, we say we have stopped the cough, but at the expense of some equally important function. The early consideration and proper treatment of cough in all irritations of the bronchial tubes as well as pulmonary structure proper is a most important one. Our patients are never comfortable while the harassing cough continues. It leads to loss of sleep, loss of appetite, loss of strength and flesh. Anxiety and worry of grave tubercular trouble makes our patients neurasthenic and plunges them deeper into the grasp of the disease.

Cough is always a manifestation of irritation and must be traced to its source to be properly treated. If it is not relieved, we well know that the irritation becomes greater, but the relief must not be at the expense of the digestive or cardio-vascular systems. The value of heroin in affections of the respiratory apparatus has been recognised by the profession since its introduction a few years ago. We have watched with interest the various preparations of this drug put upon the market—some to be condemned, some to be accepted for general use. What we need is a safe efficient preparation, whose action is definite and positive. Such preparations are hard to obtain, but the combinations with glycerine are undoubtedly the best. Glycoheroin (Smith) has recently come under my notice, and all who have used it, I think, will easily place it first among this class of preparations. A combination, as it is, of heroin with valuable expectorants and balsams, places it practically in a class by itself.

It will be found useful in any condition in which the cough is of a spasmodic character and out of proportion to the expectoration, and very serviceable in the treatment of all the various affections of the respiratory tract associated with cough. The distressing cough, the cause of many sleepless nights, I have seen relieved by one or two doses of this preparation. The effect was like magic, and a more grateful patient could not be found. Cases of asthma, in which the ordinary remedies failed totally, were relieved by glycoheroin. Excellent results are obtained in bronchitis and pulmonary irritations following ether anesthesia. Several such cases gave me a great deal of anxiety, especially in abdominal sections, but they were completely relieved by a few doses of this form of glycoheroin.

In phthisis, I have used it in combination with other remedies and find that the cough is lessened, night sweats decreased and the appetite improved. Many of our patients will be children and the greatest care must be exercised in their treatment. The opiates are invariably followed by some minor complication that will cause trouble later. The smallest child can take Smith's glycoheroin in the proportionate dose and get relief without an untoward spmptom.

Cases of whooping cough having thirty to forty paroxysms, have so improved in a few days that three or four paroxysms would be the average. In operative cases, where the wound is endangered from excessive coughing, no drug can be of more service in the treatment of irritations following ether anesthesia. We have in it a means of obtaining good results, with a certainty that is almost specific, and without any of the distressing symptoms so frequently following the use of other preparations. In not one case have I seen a bad effect following the use of this preparation, and in only one case did I see no improvement. This was a case of pulmonary edema complicating nephritis, in which I had to use more heroic treatment to get any relief whatever.

